# Mechanism of Ferroptosis and Its Role in Spinal Cord Injury

**DOI:** 10.3389/fneur.2022.926780

**Published:** 2022-06-09

**Authors:** Fei Li, Haifan Wang, Hao Chen, Jianing Guo, Xiaoqian Dang, Yi Ru, Haoyu Wang

**Affiliations:** ^1^Department of Orthopedics, The Second Affiliated Hospital of Xi'an Jiaotong University, Xi'an, China; ^2^Basic Medical Science Academy, The Air Force Medical University, Xi'an, China; ^3^State Key Laboratory of Cancer Biology, Department of Biochemistry and Molecular Biology, Basic Medical Science Academy, The Air Force Medical University, Xi'an, China

**Keywords:** spinal cord injury, regulated cell death, ferroptosis, iron homeostasis, lipid peroxidation

## Abstract

Ferroptosis is a non-necrotic form of regulated cell death (RCD) that is primarily characterized by iron-dependent membrane lipid peroxidation and is regulated by cysteine transport, glutathione synthesis, and glutathione peroxidase 4 function as well as other proteins including ferroptosis suppressor protein 1. It has been found that ferroptosis played an important role in many diseases, such as neurodegenerative diseases and ischemia-reperfusion injury. Spinal cord injury (SCI), especially traumatic SCI, is an urgent problem worldwide due to its high morbidity and mortality, as well as the destruction of functions of the human body. Various RCDs, including ferroptosis, are found in SCI. Different from necrosis, since RCD is a form of cell death regulated by various molecular mechanisms in cells, the study of the role played by RCD in SCI will contribute to a deeper understanding of the pathophysiological process, as well as the treatment and functional recovery. The present review mainly introduces the main mechanism of ferroptosis and its role in SCI, so as to provide a new idea for further exploration.

## Introduction

The central nervous system (CNS), which includes the brain and spinal cord, controls a variety of physical activities of the body. Any damage to the CNS will cause dysfunctions in the human body, including sensation, movement and even breathing and heartbeat, of which kinds of cell death play important roles.

Since 2005, the classification system has been updated by the Nomenclature Committee on Cell Death (NCCD) (1), and the latest version came out in 2018 based on the molecular mechanisms, where cell deaths have been departed into two parts, accidental cell death (ACD) and regulated cell death (RCD) (2). Since ACD has been defined as the virtually instantaneous and uncontrollable form of cell death corresponding to the physical disassembly of the plasma membrane caused by extreme physical, chemical, or mechanical cues, such as high or low temperature, acid or alkali, squeezing or shearing, RCD is identified as a form of cell death that results from the activation of one or more signal transduction modules, and hence can be pharmacologically or genetically modulated (at least kinetically and to some extent) (2).

Regulated cell death can be triggered by intracellular or extracellular disturbances, it also occurs in strictly physiological scenarios, i.e., with no relation to perturbations of homeostasis, and hence does not occur in the context of failing adaptation to stress, this particular form of RCD is called programmed cell death (PCD) (3). According to NCCD, based on the molecular mechanisms, ACD could be divided into kinds of forms, like necroptosis, pyropotosis, entotic cell death, netotic cell death, and ferroptosis.

Ferroptosis, now known as a kind of RCD, with ironically severe lipid peroxidation relying on overwhelming reactive oxygen spices (ROS) generation and imbalance in iron metabolism has been found in several diseases. It will be discussed this article the relation between ferroptosis and spinal cord injury (SCI).

## Ferroptosis

In a biological context, eukaryotic cells oxidize organic materials mainly in mitochondria and release energy. As byproducts of mitochondrial oxidative phosphorylation, ROS, on the one hand, plays important role in regular cell metabolisms, such as regulation of MAPK and PI3K signaling pathway (4). On the other, when the generation overwhelms the elimination of ROS, oxidative stress occurs, hence ROS will cause damage to nucleic acids, proteins, and lipids directly or indirectly, which, as reported, will lead to aging (5) and disease (6, 7). Additionally, excess ROS raises perturbations of iron homeostasis (8).

Iron, though in the trace, is an essential element for the human body as serving a functional constituent of various proteins, e.g., hemoglobin and cytochrome P45011.1. Since the human body is unable to excrete iron actively but with loss of red blood cells and epithelial cells (8), it is important to control iron absorption, transportation, storage, and metabolism strictly. Except for the largest portion of iron in red blood cells, most of the rest is stored in the cytosol within ferritin (9) and released as Fe^2+^ then transported *via* ferroportin (10). Notably, a cytosolic fraction of redox-active intracellular iron constitutes the labile iron pool (LIP) (11), regulated by iron homeostasis. Excess cytoplasmic iron generates ROS *via* Fenton Chemistry, leading to oxidative stress.

In 2003, a small molecule compound named erastin has been reported that it could kill engineered tumorigenic cells by inducing a non-apoptosis way (12). Several years' of studies followed suggested that this particular iron-dependent way of cell death showed the morphology of necrosis, but was mechanistically different from apoptosis or other forms of necrosis (13–15). This form of cell was named ferroptosis by Dxion, as is iron-dependent in 2012 (13).

A characteristic link of ferroptosis is the peroxidation of membrane lipids, especially phospholipids containing polyunsaturated fatty acid chain(s) (PUFA-PL) (16). As a selenoprotein, glutathione peroxidase 4 (GPX4) can directly reduce phospholipid hydroperoxides (PLOOHs) with glutathione (GSH) as a substrate, and of which the synthesis is restricted by selenoprotein synthase and selenium availability (17). GSH is the principal antioxidant of cells (18), and cysteine, the rate-limiting substrate of its biosynthesis is transferred from extracellular through system Xc^−^, the cystine/glutamate antiporter system (19). Therefore, affecting GSH synthesis, expression and activity will promote the occurrence of ferroptosis. For a long time, the cyst(e)ine/GSH/GPX4 axis has been considered the backbone of ferroptosis regulation (20), however, recent studies have found that ferroptosis suppressor protein 1 (FSP1) (21), by reducing ubiquinone (CoQ10), along with GTP cyclohydrolase 1 (GCH1)/tetrahydrobiopterin (BH4) (22) inhibited membrane lipid peroxidation from occurring.

Ferroptosis plays a role in numerous physiological and pathological processes. As embryos knockout GPX4 do not survive (23), organisms lacking GPX4 in hepatocytes (24) or neurons (25) could not succeed in living; the haploid GPX4-deficient mice have lost part of GPX4 function are more sensitive to external stimuli (26). Among many degenerative diseases, such as Alzheimer's disease (27), Parkinson's disease (28), and Huntington's disease (29) lipid peroxidation is of great importance. In a cardiac ischemic model, ferroptosis inhibitors have been shown to protect organs against ischemia-reperfusion injury (30, 31), while ferroptosis induced by conditional knockout of GPX4 induces acute renal failure (20, 32). And the possible role of ferroptosis in SCI is discussed below.

### Mechanism of Ferroptosis

#### Iron Homeostasis

Iron homeostasis is tightly regulated and could be influenced by many reasons. In human bodies, nearly 70% of iron is found in hemoglobin within red blood cells, used for oxygen transportation. Some of the rest is present in macrophages and muscle cells, and some are stored in hepatocytes (33). Though only an extremely little iron is contained in other cells, it has rather an important function, serving as a major functional component of various enzymes and proteins, such as P450 (34). Iron is absorbed into plasma in the small intestine, transported throughout the body in the form of Fe^3+^ bounding to transferrin (Tf) (35), and taken up into cells with a combination of Tf and Tf receptor (TfR) (36) and Fe^3+^ is then reduced to Fe^2+^ (37). The excess intracellular iron will be combined with ferritin in the form of Fe^3+^for storage (9), which could be re-released and utilized (38), leaving only a very small amount of intracellular iron in the redox-active form, the labile iron pool (LIP) (11, 39).

Iron homeostasis is primarily regulated by intracellular iron in a post-transcriptional modification manner by affecting the binding of iron regulatory protein 1 or 2 (IRP1 or IRP2) and iron response element (IRE), which is located in the non-coding regions of ferritin and transferrin mRNAs (40, 41). When IRP binds to the 3'UTR region of mRNA, such as TfR, it will stabilize the mRNA structure and increase its translation into protein, while binding to the 5'UTR region, such as ferritin, blocks the binding of mRNA to ribosomes thereby inhibiting gene expression (40, 42). Different from IRP1 forming a 4Fe-4S cluster (43), IRP2 will be degraded by the ubiquitin-proteasome under iron-rich conditions (44), and thus both will be unable to bind to IRE, resulting in increased ferritin synthesis and decreased TfR synthesis, leading to increased iron storage and blocked extracellular iron translocation into the cytosol. IRE is also present in the mRNA of the ferroportin-1 (FNP1) and the divalent metal transporter protein 1 (DMT1), which is subject to post-transcriptional regulation by iron (42). Under oxidative stress, ROS causes the removal of the 4Fe-4S cluster from IRP1 (45) and also inhibits iron-dependent ubiquitin degradation of IRP2 (44). Both of these enhance the binding of IRP to IRE, which increases the expression of the transferrin receptor and decreases ferritin synthesis, then finally increases intracellular iron.

#### Lipid Peroxidation

Peroxidation of PUFA-PL is a characteristic aspect of ferroptosis (16). It is believed that lipid peroxidation is divided into two pathways, spontaneous and enzyme-catalyzed. In spontaneous peroxidation, lipids are deprived of hydrogen atoms by ROS, mainly hydroxyl radical (·OH), and react rapidly with O_2_ to generate phospholipid peroxyl radical (PLOO·), which can acquire hydrogen from adjacent PUFAs thereby forming phospholipid hydroperoxides (PLOOHs) and a new PLOO-, whereupon chain reactions occur downstream. ·OH of spontaneous peroxidation can be generated by Fenton or Fenton-like reaction, and Fe^2+^ and O_2_ play key roles in the spontaneous peroxidation of PUFAs. Lipophilic antioxidants can provide electrons and neutralize atomic groups, and thus could blockchain reactions (46–48). In contrast, enzyme-catalyzed PUFA-PLs peroxidation is controlled by the lipoxygenase (LOX) family. LOXs, mainly using arachidonic acid (AA) and linoleic acid (LA) as substrates, catalyzes the production of PLOOHs from PUFA-PLs (49). Iron plays an active role in lipid peroxidation; on the one hand, Fe^2+^ from LIP is involved in spontaneous PUFA-PL peroxidation by generating ·OH through the Fenton reaction (50) and, at the same time, iron-containing LOXs mediate the enzyme-catalyzed peroxidation (51).

The precise mechanism by which peroxidized phospholipids mediate subsequent cell death remains to be investigated (52, 53). Moreover, it is inconclusive whether spontaneous peroxidation or enzymatic reactions play a dominant role in the onset of ferroptosis. It has been observed that inhibition or knockdown of LOX inhibited the occurrence of ferroptosis in some cell lines (54), implying the role of enzymatic lipid peroxidation, but this could not explain the inability of knockdown of LOX to prevent ferroptosis caused by GPX4 deletion (20).

### Regulation of Ferroptosis

#### GPX4 and GSH

Glutathione peroxidase 4 uses GSH as a substrate to reduce LOOH of peroxidized phospholipids to PLOH to remove their redox activity (18). GSH is synthesized from cysteine, glutamate, and glycine, of which cysteine is the rate-limiting substrate and mainly transported by system Xc^−^, an antiporter consisting of a catalytic subunit SLC7A11 and the chaperone subunit SLC3A2 (55). For every cystine transported into the cell, glutamate is transported out and then the cystine will be changed into cysteine with the existence of NADPH (56, 57). Both the expression and function of system Xc^−^ affect the intracellular cysteine and GSH content, and it was also the identified target protein of the first discovered ferroptosis inducer erastin (12, 58). Elevated extracellular glutamate concentrations inhibit the antiport process, which may explain the neurological toxicity of glutamate (58). Sulfasalazine and sorafenib also inhibit SLC7A11 directly or indirectly which causes the depletion of GSH (59). Activating transcription factor 4 (ATF4) and/or nuclear factor erythroid 2-related factor 2 (NRF2) are two major transcription factors that regulate stress-induced SLC7A11 expression, upregulating its transcriptional level upon stress onset, whereas p53 represses the process (55). In addition, p14ARF inhibits ATF4-mediated SLC7A11 transcription inducing ferroptosis while Cullin 2 RING E3 ligase (CRL2)-KLHDC3 E3 ubiquitin ligase complex inhibits ferroptosis by degrading p14 (60). OTUB1 (61), as well as mTORC2 (62), also regulate SLC7A11 expression and role in subsequent ferroptosis. Cellular cysteine can also be converted from methionine *via* the transsulfuration pathway, but in general, this way is low efficiency (63). Enhancement of the transsulfuration pathway is effective in suppressing ferroptosis when the antiport system is impaired (64).

Glutathione peroxidase 4 belongs to the selenoprotein family and is characterized by the presence of selenocysteine in the active center. GPX4 is the only enzyme that can reduce complex phospholipid peroxides and has a central role in triggering ferroptosis (17). RSL, an inducer of ferroptosis, binds to selenocysteine of GPX4 and inhibits its phospholipid peroxidase activity, thereby triggering ferroptosis, which could be inhibited by GPX4 overexpression (54). Inhibition of phosphoseryl-tRNA kinase (PSTK), the key enzyme in selenocysteine synthesis, inactivates GPX4 while simultaneously inhibiting GSH biosynthesis, inducing ferroptosis (65). It has been shown that over physiologic concentrations of Se upregulate GPX4 transcription, suggesting a role for Se in ferroptosis (66). Furthermore, the cystine uptaking process relied on system Xc^−^ upregulates GPX4 expression *via* activation of mTORC1 and inhibits ferroptosis (67).

#### FSP1 and CoQ10

In 2019, two groups simultaneously discovered a protein that could prevent ferroptosis in deficiency of GPX4, at first the protein was named apoptosis-inducing factor mitochondrial 2(AIFM2), and then renamed ferroptosis inhibitory protein (FSP1). FSP1 is localized to lipid membranes or lipid droplets. After N-terminal myristoylation, FSP1 can reduce CoQ10 to ubiquinol (CoQH2), which could remove lipid peroxides (21, 68). Besides, studies suggest that FSP1 could inhibit ferroptosis through an ESCRT-III-dependent membrane repair pathway in addition to CoQ10 (69), while p53 has not shown a significant role yet in the FSP1 pathway-related ferroptosis, as FSP1 shows no sensitivity to p53 agonist rubicin (68). Furthermore, it has been found that the mitochondrial dihydroorotate dehydrogenase (DHODH) can also inhibit ferroptosis independent of cytoplasmic FSP1 and GPX4 by reducing ubiquinone to ubiquinol and that the DHODH inhibitor brequinar induces ferroptosis and selectively inhibits GPX4^low^ tumor growth (70, 71).

#### Others

Acetyl coenzyme A (Acyl-CoA) synthetase 4 (ACSL4) is involved in membrane PUFA-PLs synthesis. Cells deficient in ACSL4 have shown significant resistance to RSL3-induced ferroptosis (72). In addition to transcriptional repression of SLC7A11, p53 indirectly activated the ALOX12 gene to induce ferroptosis, and this regulation was not dependent on GPX4 or ACSL4 (73). Subsequent studies found that overexpression of the phospholipase iPLA2β inhibited ALOX12-induced lipid peroxidation and suppressed FSP1 knockout-induced ferroptosis. P53 activation increased the mRNA level of iPLA2β1, and iPLA2β1 deletion did not affect embryonic development or normal tissue homeostasis (74).

**Figure 1:**
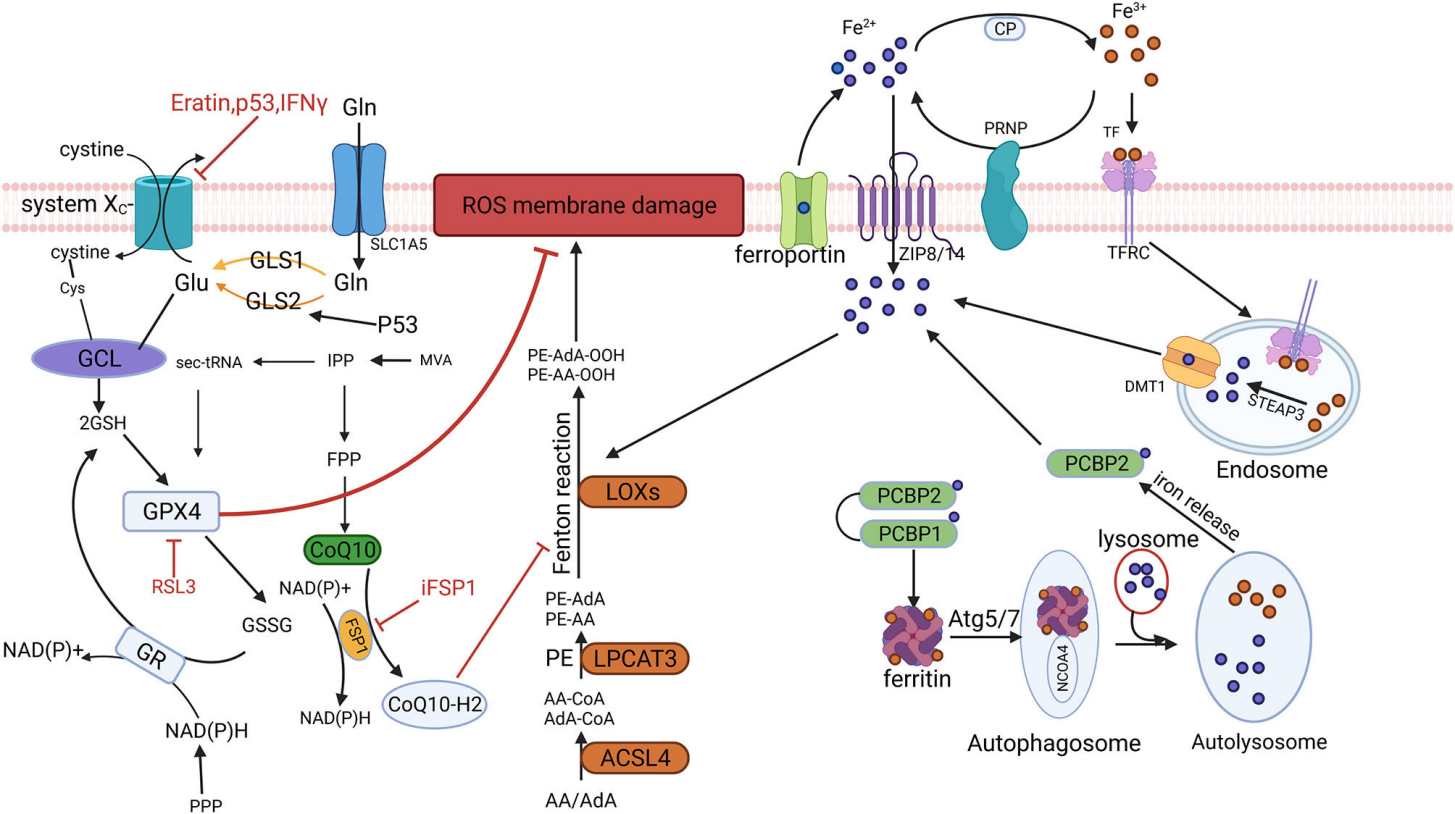
Mechanism and regulation of ferroptosis. Reactive oxygen species (ROS)-induced membrane lipid peroxidation is a key link in ferroptosis. After the extracellular Fe^3+^ is transferred into the cell, it is reduced to Fe^2+^, and the free Fe^2+^ generates ROS through the Fenton reaction, which mediates the occurrence of lipid peroxidation. Cystine is transported into the cell by System Xc^−^ and participates in the synthesis of glutathione (GSH). Glutathione peroxidase 4 (GPX4) reduces peroxidized membrane lipids using GSH as substrates. Ferroptosis inhibitory protein (FSP1) scavenges peroxidized membrane lipids and inhibits ferroptosis by reducing ubiquinone (CoQ10) to ubiquinol (CoQH2). ROS, reactive oxygen species; GSH, glutathione; GPX4, glutathione peroxidase 4; FSP1, ferroptosis inhibitory protein; CoQ10, ubiquinone; CoQH2, ubiquinol.

Genome-wide activation screens identified guanosine triphosphate cyclohydrolase 1 (GCH1), the rate-limiting enzyme of tetrahydrobiopterin (BH4) biosynthesis, as a potent antagonist of ferroptosis, and overexpression of GCH1 enhanced the synthesis of BH4 while inhibiting the onset of ferroptosis independently of the GSH pathway. BH4 protects 2PUFA-PLs from degradation on the one hand and promotes the synthesis of CoQ10 on the other (22). Other studies also found that RSL3 triggered the onset of ferroptosis by activating the NF-κB pathway (75). Besides, the murine double minute 2—murine double minute X complex (MDM2-MDMX complex), a negative regulator of p53, promotes ferroptosis mediated by peroxisome proliferator-activated receptor α (PPARα) in the presence or absence of p53 (76) (Figure 1).

## Ferroptosis in SCI

Spinal cord injury refers to various factors leading to damage to the spinal cord, resulting in motor, sensory, and other functional impairments in the corresponding stages. SCI can be divided into traumatic SCI and non-traumatic SCI depending on the etiology. Non-traumatic SCI usually occurs with other primary diseases such as tumors or spinal disc herniation. Traumatic SCI is often caused by the impact of strong external forces, such as traffic injuries (77). According to reports, the overall global incidence of traumatic SCI is 10.5 per 100,000 people, that is, about 768,473 traumatic SCI occur globally each year, of which 48.8% require surgical intervention (78). Mortality from SCI is related to a variety of factors, with higher injured segment, older age, and greater violence leading to increased odds of death (77).

**Figure 2:**
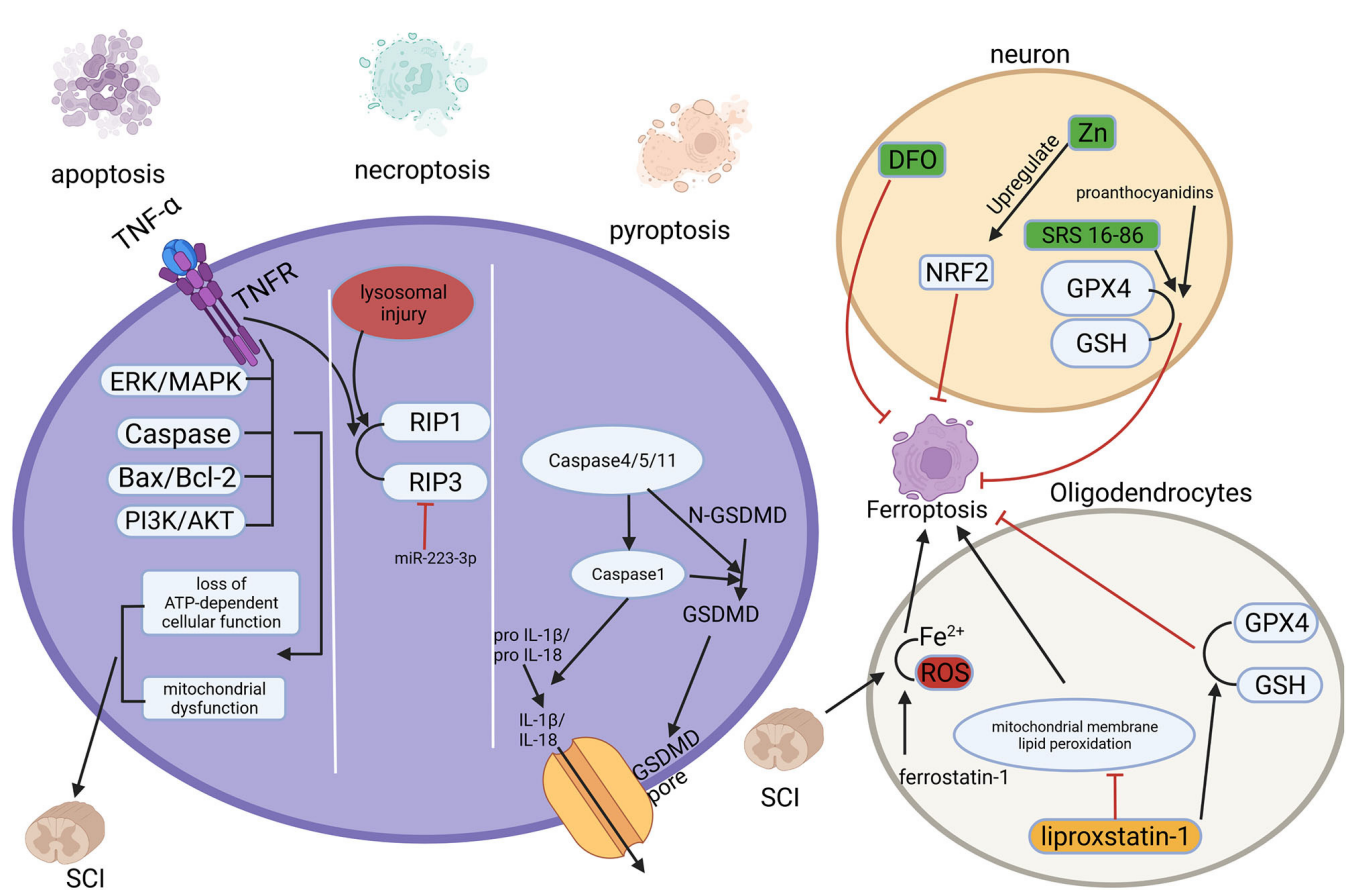
Multiple regulated cell deaths (RCDs) play a role in spinal cord injury (SCI). RCD, regulated cell death; SCI, spinal cord injury.

Pathophysiologically, SCI can be divided into primary and secondary injuries. Primary injury is dominated by violence-induced hemorrhage and cellular necrosis; in the secondary injury stage, cells under regulation by pro-apoptotic and other factors, show dysfunction or death, along with inflammatory cell infiltration and cellular swelling. RCD plays a considerable role in this stage, and several types of RCD in traumatic SCI are discussed below (77) (Figure 2).

### The Role of RCD in SCI

#### Apoptosis

Apoptosis is the most common form of RCD and can be considered programmed self-kill (79). It can be initiated by endogenous factors such as mitochondria or exogenous factors such as inflammatory factors (80, 81). The loss of function due to primary SCI is primarily a direct result of cellular necrosis including neurons and glial cells caused by external forces. Subsequent progressive enlargement of the injury area and progressive loss of function are associated with apoptosis (82). Apoptosis of neurons, astrocytes, oligodendrocytes, and microglia have been observed within hours to weeks after the injury happened (83). Mitochondria perform a crucial part in apoptosis, and the distinguishing features of the secondary phase of SCI are mitochondrial dysfunction and loss of ATP-dependent cellular function (84). In addition, apoptosis-related proteins and pathways, like the caspase protein family (85), Bax/Bcl-2 (86), TNF-α (87, 88), as well as PI3K/AKT (89, 90), and ERK/MAPK pathway (89, 91) play significant roles in the secondary phase of SCI.

#### Autophagy

Autophagy is a non-apoptotic form of RCD that degrades and recycles damaged organelles, and useless proteins through autophagosome-lysosome maintaining cellular viability and homeostasis (92, 93). Autophagy can be classified as macroautophagy, microautophagy, and molecular chaperone-mediated autophagy (94). During the process of autophagy, bilayer membrane autophagosomes wrap damaged organelles or proteins then fuse with lysosomes, and under an acidic environment, these contents are degraded (95). Autophagy occurs during the secondary SCI, but the specific role of autophagy is controversial. Some studies suggest that after SCI, upregulating autophagy inhibits the initiation of apoptosis and promotes post-injury repair of the nervous system (96, 97). However, others suggest that inhibition of autophagy after SCI may provide protection *in vivo* (98, 99). These seem to imply that the level and the role of autophagy after SCI depends specifically on the severity and location of the injury (95).

#### Necroptosis

Necroptosis is a regulated form of necrotic cell death with morphological manifestations of necrosis and lacking typical apoptotic features (2). Necroptosis is mainly mediated by receptor-interacting protein (RIP) 1 and 3 kinases, and the most intensively studied form of initiation is the binding of tumor necrosis factor-α (TNF-α) to the receptor (100). Necroptosis was found to exist in neurons and glial cells after SCI, and upregulation of miRNA-223-3p inhibits necroptosis targeting RIP3 (101), while lysosomal injury leads to accumulation of RIPK3 and 1 enhances it (102). Moreover, quercetin (103) and dabrafenib (104) have been found to inhibit necroptosis after SCI attenuate SCI and promote recovery.

#### Pyroptosis

Pyroptosis, known as inflammatory necrosis of cells, is a new type of RCD (2), mainly regulated by activation of caspase-1, caspase-4/5/11, as well as the cleavage of the pore-forming protein GSDMD and the release of inflammatory factors IL-1β and IL-18 (105, 106). Pyroptosis plays a key role in infectious diseases and SCI, as secondary SCI is accompanied by the release of large amounts of inflammatory factors (107). In a rat model of SCI, the levels of pro-inflammatory cytokines and the expression of pyroptosis-related proteins such as caspase-1 and GSDMD were significantly increased (108). For patients with SCI, the expression of GSDMD in peripheral blood is raised and positively correlated with the degree of injury (109). It was found that betulinic acid inhibits pyroptosis by enhancing autophagy through the AMPK-mTOR-TFEB pathway (110). And kaempferol pretreatment attenuates neuroinflammation by inhibiting pyroptosis after secondary SCI by inhibiting the MAPKs-NFκB pathway (111).

#### Ferroptosis

In traumatic SCI, the primary injury immediately induces cell damage and triggers a continuous cascade of secondary injuries that induce ischemia, inflammation, and cell death (77). Ferroptosis occurs during SCI with increased iron and ROS accumulation at the injury site (112), and excessive lipid peroxidation (113, 114) observed in adult mouse models that conditional ablation of Gpx4 in neurons can induce degeneration of motor neurons. In fact, it is well known that excitatory toxicity induced by glutamate accumulation precedes neuronal death and astrocyte reuptake failure, followed by ferroptosis and secondary injury after SCI (77). The role of ferroptosis in SCI requires further investigation.

### Ferroptosis-Related SCI Studies

**Figure 3:**
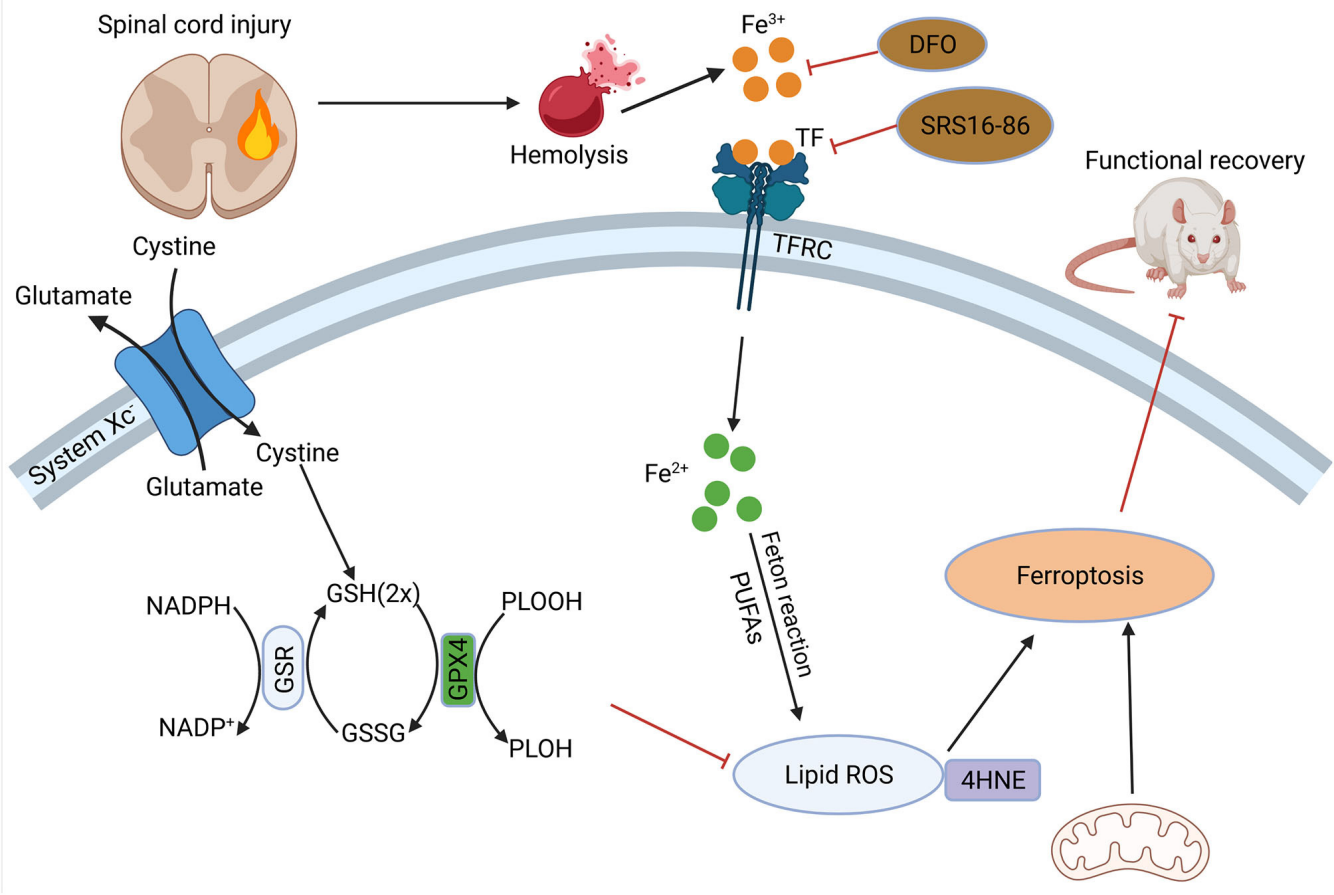
Role of ferroptosis in SCI. Hemorrhage after SCI increases iron deposition and induces ferroptosis. DFO can inhibit ferroptosis after SCI and promote the recovery of nerve function. SCI, spinal cord injury; DFO, desferrioxamine.

There is no doubt the understanding of whether ferroptosis occurs in spinal cord injury and the specific role it plays in the pathophysiological process is crucial to the study of ferroptosis-related SCI and potential therapeutic directions (Figure 3). After primary spinal cord injury occurs, local changes such as hemorrhage and inflammatory cell infiltration occur. It was found that in SCI patients and animal models, iron deposition increased, triggering the accumulation of ROS and leading to motor neuron death. Iron chelators, ROS, and ferroptosis inhibitors reduce iron deposition-induced ferroptosis and promote motor recovery. At the same time, microglia activate and secrete large amounts of NO, causing iron overload in the motor cortex (115).

In 2019, Yao, X.et.al found that desferrioxamine(DFO), a ferroptosis inhibitor, could protect neurons, and promote motor function recovery. In 90 SCI model rats they established, the experimental group was intraperitoneally injected with DFO, and it was found that the DFO group had better functional recovery, lower iron concentration, and significantly higher expression of GPX4 and GSH. Meanwhile, DFO increased neuronal survival and inhibited glial proliferation (116). And another study confirmed the protection of DFO was established on inhibition of erastin-induced ferroptosis in neuronal cells (117). The modulation of ferroptosis-related pathways after spinal cord injury is of great value for the recovery and reconstruction of spinal cord function. In a recent study, zinc showed its ability of inhibit ferroptosis occurrence by activating the NRF2 pathway, which had a positive effect on the functional recovery of the nervous system (118). Other studies have shown that proanthocyanidins and the ferroptosis inhibitor SRS 16-86 can upregulate GSH and GPX4 expression and function after SCI, thus enhancing neuronal cell resistance to membrane phospholipid peroxidation and inhibiting the occurrence of ferroptosis, thereby promoting the recovery of motor function after spinal cord injury (119, 120).

In addition, the white matter of the spinal cord, as a signal conducting way, plays an important role in the function of the nervous system, and damage to and repair of the white matter is an important component of functional recovery after SCI. Oligodendrocytes are the main components of white matter formation and biological functions. In a recent study, it was found that in a rat model, ferrostatin-1 attenuated iron and ROS deposition in oligodendrocytes, inhibited their ferroptosis, attenuated white matter injury and promoted nerve conduction (121). In a similar study, it was revealed that in a model of RSL-3-induced ferroptosis in oligodendrocytes, liproxstatin-1 depressed mitochondrial membrane lipid peroxidation, restored GSH and GPX4 expression, thus showing stronger effect of blocking ferroptosis than DFO (122). Therefore, the study of ferroptosis-related genes and proteins will be an important strategy to attenuate neuronal and oligodendrocyte injury and promote the recovery of neurological function after SCI. Our previous work showed that Mettl14-mediated m6A modification inhibited RASD1 and induced the apoptosis of spinal cord neurons in SCI by promoting the transformation of pri-miR-375 to mature miR-375 (123). However, it is still unclear of the iconic molecules of ferroptosis yet, the study of ferroptosis mechanism is still not deep enough, and the role of ferroptosis in SCI is still in the mist, the studies with patient and clinical proximity are relatively rare, which are the urgent issues in this field.

## Conclusion

Ferroptosis is a kind of RCD characterized by membrane lipid peroxidation, in which GPX and GSH, FSP1 and CoQ10, and other molecular mechanisms play huge roles. Including ferroptosis, various RCDs, such as apoptosis, autophagy, necroptosis, and pyroptosis, play their roles in secondary SCI, but the specific mechanisms and clinical applications remain to be further studied.

## Author Contributions

FL performed the literature search, summary, and wrote the manuscript. HFW was responsible for the literature search and visualization presentation. HC and JG were assigned to the literature search. XD provided technical guidance. YR and HYW were responsible for the concept, critical review, and revision of the manuscript. All authors have read and approved the final manuscript.

## Conflict of Interest

The authors declare that the research was conducted in the absence of any commercial or financial relationships that could be construed as a potential conflict of interest.

## Publisher's Note

All claims expressed in this article are solely those of the authors and do not necessarily represent those of their affiliated organizations, or those of the publisher, the editors and the reviewers. Any product that may be evaluated in this article, or claim that may be made by its manufacturer, is not guaranteed or endorsed by the publisher.
